# Anorexia nervosa and gastrointestinal diseases: A multivariable Mendelian randomization study

**DOI:** 10.1097/MD.0000000000044503

**Published:** 2025-09-19

**Authors:** Cheng Pu

**Affiliations:** a School of Martial Arts, Shanghai University of Sport, Shanghai, P.R. China.

**Keywords:** acute gastritis, anorexia, Crohn disease, gastrointestinal diseases, Mendelian randomization

## Abstract

Research on the associations between anorexia nervosa (AN) and gastrointestinal (GI) diseases is limited, and the causality and underlying directionality remain unclear. Therefore, we aimed to elucidate the causal associations between AN and the risk of GI diseases through Mendelian randomization (MR) analyses. Summary statistics for AN and 24 GI diseases were obtained from the Psychiatric Genomics Consortium and the public available genome-wide association study databases. Single nucleotide polymorphisms associated with AN and GI diseases at the genome-wide significance level were selected as instrumental variables to perform bidirectional 2-sample MR analyses. Additionally, we performed multivariable MR analyses adjusting for smoking and alcohol consumption. The inverse-variance weighted method was used as the primary MR method, and a series of sensitivity analyses were applied to assess heterogeneity and pleiotropy. Genetically predicted AN showed nominally significant association with higher risk of acute gastritis (OR = 1.593, 95% CI: 1.081–2.347; *P* = .019) and Crohn disease (OR = 1.001, 95% CI: 1.00008–1.003; *P* = .038). Reverse MR analyses found a nominally significant positive association between gastric cancer and AN, but no reverse causality was observed for the others. After adjustment for smoking and alcohol consumption, the nominally significant associations of AN with acute gastritis and Crohn disease remained, while the association between gastric cancer and AN became nonsignificant. Our study provides preliminary genetic evidence supporting potential causal associations between AN and specific GI diseases and highlights priority targets for future investigation. Further studies are necessitated to validate these findings and elucidate the underlying mechanisms.

## 1. Introduction

Characterized by nutritional restrictions and low body weight, anorexia nervosa (AN) is one of the most prevalent types of eating disorder with serious repercussions,^[1]^ including increased morbidity and mortality^[2]^ and higher risk of suicide.^[3]^ Moreover, AN is commonly accompanied by a spectrum of medical complications affecting all organs and systems, with gastrointestinal disorders particularly common,^[4,5]^ thus complicating its clinical management and impairing health status and life quality.^[6]^

Studies have increasingly recognized the high comorbidity and complex associations between gastrointestinal (GI) diseases and psychiatric disorders, such as depression and anxiety,^[7–10]^ but research on the associations between AN and GI diseases is limited. And intriguingly, such associations are not unidirectional. AN may pose a risk factor for GI symptoms, such as irritable bowel syndrome (IBS) and inflammatory bowel disease. Conversely, GI diseases could also be risk factors for AN or exacerbate its symptoms.^[11–13]^ Therefore, the causal relationships between AN and GI diseases and the underlying directionality remain largely unclear, warranting further investigation.^[14,15]^

However, most relevant research to date has been based on observational studies, primarily consisting of case reports and general studies on autoimmune diseases in people with eating disorders.^[16–18]^ Such studies are inevitably susceptible to confounding bias and reverse causality, and thus have limited capacity to establish causality. And conventional randomized controlled trials are confronted with high costs, ethical issues and other constraints. Mendelian randomization (MR) has become an alternative approach to establishing causality. It utilizes genetic variants associated with exposure, i.e., single nucleotide polymorphisms (SNPs), as instrumental variables (IVs) to infer the causal association of exposure with outcome. Recently, the Psychiatric Genomics Consortium (PGC) has identified 8 AN-associated genome-wide significant loci,^[19]^ presenting us with an unprecedented opportunity to investigate the causality between AN and GI diseases.

Furthermore, multivariable MR (MVMR) is a recent extension to MR that utilizes SNPs associated with several, possibly related exposures to assess the independent direct effects of each exposure on 1 single outcome.^[20]^ By estimating associations conditionally on potential confounders, it can reduce potential pleiotropic effects. AN has been reported to be correlated with smoking and alcohol consumption,^[21–23]^ which, in turn, have been shown to be risk factors for multiple GI diseases.^[24–26]^ Therefore, these factors could be potential confounders in the associations between AN and GI diseases.

Here in our study, we performed bidirectional univariable MR study to examine the causal associations between genetically predicted AN and 24 GI diseases. Furthermore, we further performed multivariable MR analyses, which would contribute to more comprehensive insights into the causal structure underlying the identified associations between AN and GI diseases.

## 2. Methods

### 2.1. Study design

**Figure 1.**
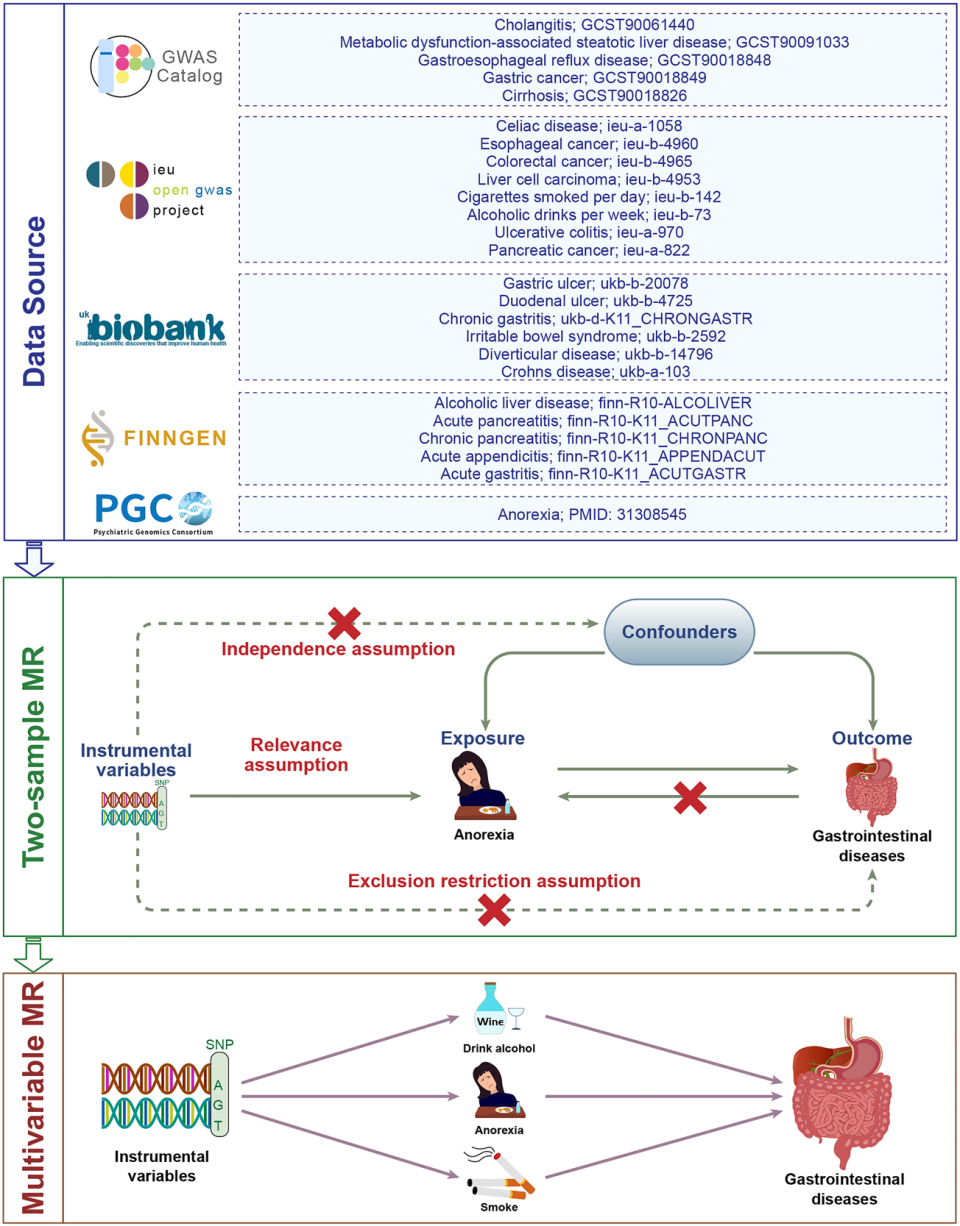
Research flowchart.

Our research flowchart is shown in Figure 1. First, we used AN as exposure and 24 GI diseases as outcomes to perform univariable MR (UVMR) analyses. Next, the exposure and outcome were switched for reverse MR analyses to avoid possible reverse causality. Subsequently, we performed MVMR analyses accounting for potential confounding effects of smoking and alcohol consumption. The MR analysis is based on 3 basic assumptions: (i) IVs are strongly associated with exposure; (ii) IVs are independent of confounding factors; (iii) IVs only affects the outcome through exposure.

### 2.2. Data sources

To minimize population stratification bias, the population was mainly of European ancestry.

Summary statistics for AN were extracted from the PGC (https://www.med.unc.edu/pgc/results-and-downloads/) genome-wide association study (GWAS) meta-analysis by Watson et al, which incorporated 16,992 cases and 55,525 controls of European ancestry from 33 cohorts.^[19]^ Case definition included a lifetime diagnosis of AN (restricting or binge–purge subtype) or lifetime eating disorders “not otherwise specified” AN subtype according to standardized criteria, including Diagnostic and Statistical Manual of Mental Disorders Third Edition-Revised (DSM-III-R), DSM Fourth Edition (DSM-IV), International Classification of Diseases Eighth Edition (ICD-8), ICD-9, or ICD-10.

Summary-level data for 24 GI diseases was primarily from the UK Biobank and the FinnGen study, as well as large consortium such as the International Inflammatory Bowel Disease Genetics Consortium (IIBDGC) and the Pancreatic Cancer Cohort Consortium (PanScan). The GI diseases can be divided into 4 categories according to the site of occurrence: upper GI diseases (gastroesophageal reflux disease, esophageal cancer, gastric ulcer, duodenal ulcer, acute gastritis, chronic gastritis, and gastric cancer); lower GI diseases (IBS, celiac disease, diverticular disease, Crohn disease, ulcerative colitis, and colorectal cancer); hepatobiliary and pancreatic diseases (metabolic dysfunction-associated steatotic liver disease, alcoholic liver disease, cirrhosis, liver cancer, cholangitis, cholecystitis, cholelithiasis, acute pancreatitis, chronic pancreatitis, and pancreatic cancer); and other (acute appendicitis). Detailed information is summarized in Table S1, Supplemental Digital Content, https://links.lww.com/MD/Q67.

GWAS summary data for smoking and alcohol consumption was obtained from the GWAS and Sequencing Consortium of Alcohol and Nicotine use (GSCAN).^[27]^

### 2.3. Instrumental variable selection

**Figure 2.**
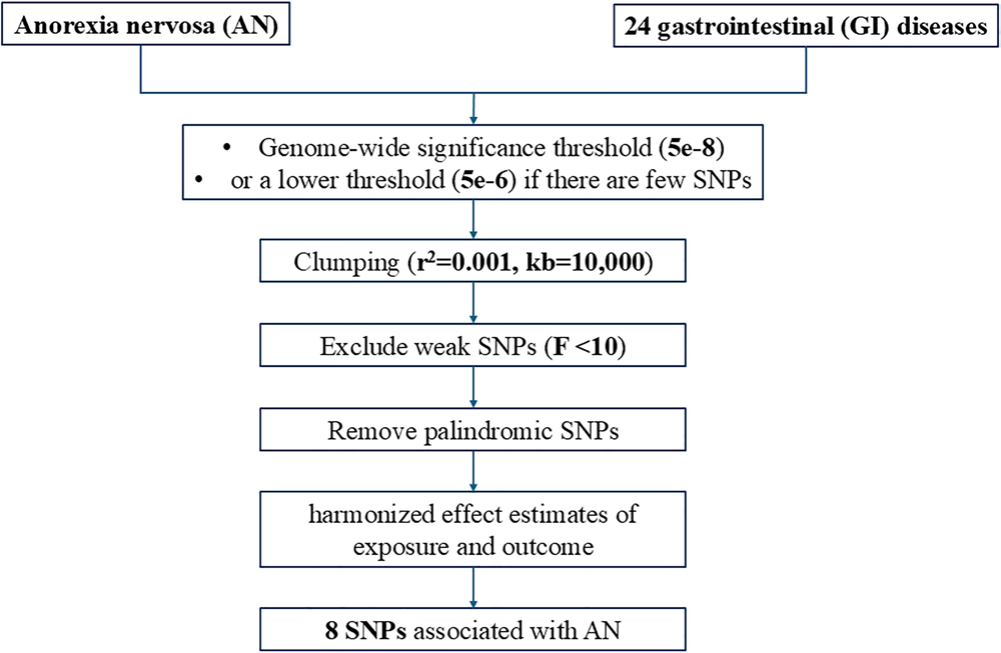
SNP selection process. SNP = single nucleotide polymorphism.

The following quality control criteria were used to select IVs (Fig. 2). First, SNPs at the genome-wide significance threshold (*P* < 5 × 10^−8^) were selected as eligible IVs for the exposure. In case of few SNPs, we used a lower threshold (*P* < 5 × 10^−6^) instead. Then, SNPs were clumped (*r*^2^ = 0.001, kb = 10,000) to exclude strong linkage disequilibrium. Next, we calculated *F* statistics and removed weak IVs with *F* statistics <10 to avoid weak instrumental variable bias. Moreover, we harmonized effect estimates of exposure and outcome, and removed palindromic SNPs.

### 2.4. MR analysis

To assess the causal effects of AN on GI diseases, we performed 2-sample UVMR analyses. The MR analysis was mainly based on inverse-variance weighted (IVW) method. Since IVW assumes that all IVs are valid and independent, it is susceptible to the effects of instrumental variable pleiotropy and heterogeneity. Therefore, we used 3 additional methods as complements for causal inference, including MR-Egger regression, weighted median, and weighted mode, and their consistent estimate directions can improve the robustness of IVW results. The weighted median method provides valid estimates when more than 50% of the SNPs are valid. The MR-Egger can provide estimates of the causal effect after adjusting for horizontal pleiotropy.^[28]^ The weighted mode method has less strict assumptions but is less statistically powerful. Additionally, when the number of SNPs was < 2, the Wald ratio method was used to calculate MR estimate.

To assess the causal effects of GI diseases on AN, we conducted reverse MR analysis with GI diseases as exposures and AN as outcome. The analysis method and process were consistent with those in the foregoing MR analysis.

Considering that smoking and alcohol are also associated with AN and GI diseases, their confounding effects should be noted in previously identified associations, which could not be fully reflected by UVMR. Therefore, to determine whether AN acted directly on GI diseases and vice versa, we further conducted MVMR analyses to estimate their direct effects independent of smoking and alcohol consumption.

### 2.5. Sensitivity analysis

To verify the robustness of MR results, we conducted an array of sensitivity analyses. Cochran Q test and funnel plots were used to assess heterogeneity among SNP estimates. Furthermore, MR-Egger intercept test and MR pleiotropy residual sum and outlier (MR-PRESSO) were utilized to identify potential horizontal pleiotropy and significant outliers. To visualize MR results, we also presented forest plots, scatter plots, and funnel plots.

### 2.6. Statistical analysis

MR analyses were performed using the “TwoSampleMR” (version 0.5.6) package in R software (version 4.3.2). All estimates were expressed as odds ratio (OR) with 95% confidence interval (CI) per 1-SD of genetically predicted exposure liability. The IVW method served as the primary estimator, complemented by MR‑Egger, weighted median, and weighted mode. To address multiple testing across the 24 GI outcomes in the primary IVW analysis, we calculated false discovery rate (FDR)–adjusted q-values using the Benjamini–Hochberg procedure. Nominal significance was defined as *P* < .05, and FDR significance as q < 0.05. Given the exploratory nature of this analysis, primary in-text interpretations were based on nominal signals, with FDR-adjusted results reported in full to ensure transparency.

## 3. Results

### 3.1. Causal effects of AN on GI diseases

After stringent quality control criteria (*P* < 5 × 10^−8^, *r*^2^ < 0.001, kb < 10,000), 8 SNPs significantly and independently associated with AN were identified as IVs. And their *F* statistics were all >10, indicating no weak instrument bias (Table S2, Supplemental Digital Content, https://links.lww.com/MD/Q67).

**Figure 3.**
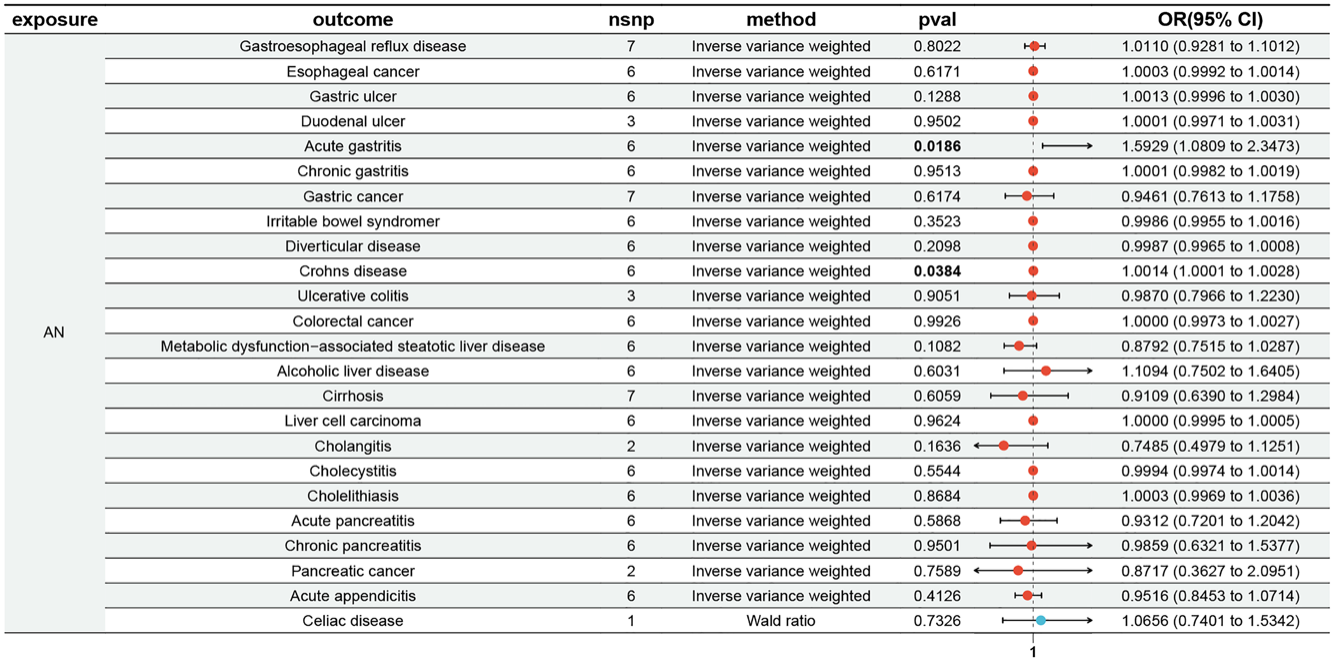
Univariable Mendelian randomization results for the associations between anorexia nervosa and gastrointestinal diseases.

UVMR analyses revealed that genetically predicted AN was nominally associated with 2 of the 24 GI diseases (1 upper GI disease and 1 lower GI disease): acute gastritis (OR = 1.593, 95% CI: 1.081–2.347; *P* = .019) and Crohn disease (OR = 1.001, 95% CI: 1.00008–1.003; *P* = .038). Across the 24 outcomes, none of the IVW associations survived FDR correction (all *q*‑values >0.05; Table S3, Supplemental Digital Content, https://links.lww.com/MD/Q67). IVW and 3 additional methods all showed consistent directions in each association, supporting robustness at the nominal level. However, no other outcomes showed nominal causal associations (Fig. 3; Table S3, Supplemental Digital Content, https://links.lww.com/MD/Q67).

Cochran *Q* test indicated that all *P*-values were >.05, indicating the absence of heterogeneity, which was further corroborated by the results of funnel plots. MR-Egger intercept tests showed that all *P*-values were >.05, indicating no horizontal pleiotropic effects. MR-PRESSO identified no significant outlier (Table S4, Supplemental Digital Content, https://links.lww.com/MD/Q67).

### 3.2. Reverse MR analysis

In the reverse MR analyses, 24 GI diseases were used as exposures and AN as outcome. The number of SNPs identified for each GI diseases ranged from 2 to 88. The results showed that only genetically predicted gastric cancer was nominally associated with AN (OR = 1.128, 95% CI: 1.004–1.267; *P* = .043). There was no significant causal association between the other 23 GI diseases and AN (Table S5, Supplemental Digital Content, https://links.lww.com/MD/Q67).

Cochran *Q* test suggested mild heterogeneity in ulcerative colitis (*P* = .00499), but found no heterogeneity in the other GI diseases (*P* >.05). MR-Egger intercept test indicated the absence of pleiotropy (*P* >.05). MR-PRESSO test identified no significant outlier.

### 3.3. MVMR analysis

IVs used in the MVMR models are shown in Table S6, Supplemental Digital Content, https://links.lww.com/MD/Q67. After statistically adjusting for genetically predicted smoking and alcohol consumption, the causal association of AN with acute gastritis attenuated slightly but remained nominally significant (OR = 1.511, 95% CI: 1.005–2.271; *P* = .047). The causal association of AN with Crohn disease also remained nominally significant after adjustment (OR = 1.002, 95% CI: 1.0003–1.003; *P* = .019), which further strengthened the robustness of our results. However, after adjustment for smoking and alcohol consumption, the association of gastric cancer with AN was not nominally significant (OR = 1.075, 95% CI: 0.917–1.261; *P* = .373) (Table S7, Supplemental Digital Content, https://links.lww.com/MD/Q67).

## 4. Discussion

To our knowledge, this is the first MR study to comprehensively investigate the causal associations between AN and 24 GI diseases. In the bidirectional UVMR analyses, we found that out of 24 GI diseases outcomes, genetic liability to AN was nominally associated with an increased risk of acute gastritis and Crohn disease. Reverse MR analyses found a nominally significant association between gastric cancer and AN, but no reverse causality was observed for the other 23 GI diseases. In MVMR analyses, the causal associations of AN with acute gastritis and Crohn disease remained nominally significant after adjusting for smoking and alcohol consumption, while the association between gastric cancer and AN became nonsignificant. This suggests that the observed signals for acute gastritis and Crohn disease are robust to these specific confounders and warrant further investigation as priority hypotheses.

In line with our findings, the association between AN and the risk of Crohn disease has been extensively reported in previous observational studies. A Denmark study based on a large, population-based sample found a positive association between a prior diagnosis of AN and later risk of Crohn disease with a hazard ratio of 1.60 (95% CI:1.04–2.46), and no reverse association was observed.^[29]^ Similarly, 2 cohort studies conducted respectively in UK and Finland reported an increased risk of Crohn disease in patients with AN with an OR of 2.3 and 1.8.^[17,30]^ In contrast to our findings, a genome‐wide association study identified no correlation between AN and Crohn disease.^[31]^ Such inconsistent results observed may be ascribed to its relatively limited sample size of AN and residual confounding. Our study used larger GWAS data and more strict screening criteria to provide compelling genetic evidence for the causal link between AN and Crohn disease, which corroborated and extended the evidence that AN may play a causal role in increasing the risk of Crohn disease.

Regarding acute gastritis, there are few studies investigating the associations between AN and risk of acute gastritis. However, numerous studies have demonstrated that patients with AN have overall delayed gastric emptying and deranged gastric motor function disorders,^[32–34]^ which in turn is well correlated with gastritis.^[35,36]^ Additionally, abnormal secretion of gastric acid induced by AN may contribute to impaired gastric mucosal barrier function and mucosal inflammation, thus increasing the risk of gastritis.^[37,38]^ The present study is indicative of a potential causal link between AN and acute gastritis, suggesting that AN may serve as a risk factor for acute gastritis. This novel finding warrants further investigation and confirmation by future studies.

AN is also a common symptom in cancer, affecting almost half of patients diagnosed with cancer,^[39]^ and is frequently seen in gastric malignancies,^[40,41]^ which is consistent with our finding of the associations between gastric cancer and AN. The development of AN in GI caners is a multifactorial process, involving hormones, neuropeptides, and cytokines. There is evidence that many cytokines produced by tumor and immune cells can interact with neuropeptides and mediate cancer anorexia, which may explain the association between gastric cancer and AN.^[42]^ For example, cytokines can mimic leptin signaling and suppress orexigenic ghrelin and neuropeptide Y (NPY) signaling to affect energy homeostasis and induce anorexigenic effect in cancer.^[43,44]^

Considering that smoking and alcohol are closely associated with AN and GI diseases,^[21–26]^ we performed MVMR analyses to account for their potential confounding effects in previously identified associations. After the adjustment for smoking and alcohol consumption, some causal associations attenuated or became not significant, which demonstrated that smoking and alcohol had confounding effects in these associations. Unfortunately, however, in our MVMR, we did not adjust for smoking and alcohol consumption in the nonsignificant causal associations, so some other potential associations may be missed.

In contrast to our results, a few studies have noted associations between AN and other GI diseases. Cohort studies suggested that patients previously hospitalized with AN were at higher risk of esophageal cancer.^[45,46]^ Several studies have identified bidirectional associations between AN and celiac disease, and ORs for celiac disease in those previously diagnosed with AN range from 2.18 to 3.08.^[17,47]^ The study by Wotton et al also suggested an increased risk of ulcerative colitis in patients with AN.^[17]^ Associations have been found between AN and the risk of IBS.^[48,49]^ Additionally, elevated aminotransferases in AN could induce liver cell damage and hepatitis.^[50,51]^ However, our MR analysis demonstrated no causal associations for these GI diseases. Such difference from observational studies may be due to their limited sample size, surveillance bias, and potential confounders.

Genetic correlations between AN and several metabolic and psychiatric traits have encouraged a reconceptualization of AN as a metabolic and psychiatric disorder.^[52–54]^ And several biological mechanisms, including neuroendocrine pathways, immune system, and autonomic nervous system, may be involved in the associations between AN and GI diseases. One of the most important underlying mechanisms for such correlations is the gut-brain-axis, which is characterized by bidirectional interactions between gastrointestinal tract and the central nervous system, and has an important role in linking psychiatric disorders to GI diseases.^[55]^ Of note is the prominent role of gut microbiota as key regulators in the gut microbiota-gut-brain axis via immune, endocrine and neural pathways.^[56–58]^ Previous studies have provided evidence of gut microbiota dysbiosis and reduced alpha diversity, and increased intestinal permeability in AN,^[59–62]^ which can impair intestinal barrier function and cause inflammation, leading to gastrointestinal disorders and disease states.^[63,64]^ Additionally, AN has been shown to be associated with dysregulated immune system and chronic low-grade inflammation, as strongly suggested by elevated levels of proinflammatory cytokines and other markers of inflammation.^[65–67]^ Moreover, autonomic nervous system dysfunction observed in AN^[68,69]^ can also lead to dysfunction of gastrointestinal tract through the enteric nervous system.^[70,71]^ Other factors such as vomiting, diuretic abuse, laxative abuse or psychological factors may also play a role in increasing the risk of GI diseases.

Our study has several major strengths. Firstly, we, for the first time, systematically assessed the causal relationship between AN and 24 GI diseases. Based on the MR design, our analysis minimized the risk of reverse causality bias and confounding bias caused by environmental factors. Secondly, the use of large-scale GWAS data in our MR analysis improved the statistical power to infer causal effects. Additionally, the consistency of multiple methods and tests enhanced the reliability and robustness of our findings.

However, several limitations need to be noted in our study. Firstly, since our GWAS instruments and outcomes were primarily derived from European-ancestry cohorts, the generalizability of our findings to other ancestries may be limited. Cross-ancestry differences in allele frequencies and linkage disequilibrium can alter instrument strength and causal estimates, and ancestry-specific pleiotropic pathways and exposure distributions (e.g., smoking and alcohol) may further modify effects. Replication in non-European cohorts, trans-ethnic MR analyses, and methods that quantify ancestral heterogeneity between populations such as MR-MEGA are warranted to validate and refine these findings. Secondly, since the GWAS data used was publicly available, detailed information on participants could not be obtained for further subgroup analysis, such as age and gender stratification. Thirdly, given the strict IV selection criteria, the limited number of SNPs available for AN might introduce biases in the results. Lastly, despite employing multiple analytical methods and tests, there is no guarantee that potential pleiotropy had been completely eliminated. Notably, while exploratory analyses identified nominal signals, none of the AN→GI associations survived FDR correction across the 24 outcomes, which is likely due to the modest genetic instrument for AN and the consequently limited statistical power to detect causal effects of a likely modest magnitude. Therefore, these findings should be interpreted with caution and regarded as preliminary until replicated in larger, ideally multi-ancestry datasets with greater power.

## 5. Conclusions

To conclude, our study provided preliminary genetic evidence of potential causal associations between AN and higher risks of acute gastritis and Crohn disease, as well as a nominal reverse association between gastric cancer and the risk of AN. Further studies with laboratory data and longitudinal observations are warranted to validate these findings and elucidate the underlying mechanisms, which may shed light on more effective reciprocal prevention and management of AN and GI diseases, and improve patient outcomes.

## Acknowledgments

We would like to express our gratitude to the PGC, the UK Biobank, the FinnGen study, the IIBDGC, the Pancreatic Cancer Cohort Consortium (PanScan), and the GWAS and Sequencing Consortium of Alcohol and Nicotine Use (GSCAN) for sharing data, and to all the researchers and participants involved in their studies.

## Author contributions

**Conceptualization:** Cheng Pu.

**Data curation:** Cheng Pu.

**Formal analysis:** Cheng Pu.

**Investigation:** Cheng Pu.

**Methodology:** Cheng Pu.

**Software:** Cheng Pu.

**Validation:** Cheng Pu.

**Visualization:** Cheng Pu.

**Writing – original draft:** Cheng Pu.

**Writing – review & editing:** Cheng Pu.

## Supplementary Material


